# Yoga as an Adjunct Intervention for Chemotherapy-Induced Peripheral Neuropathy: Evidence, Challenges, and Future Directions

**DOI:** 10.7759/cureus.84106

**Published:** 2025-05-14

**Authors:** Selvaraj Giridharan, Mrunmai Godbole

**Affiliations:** 1 Oncology, Tawam Hospital, Al Ain, ARE; 2 Yoga, Jayawant Shikshan Prasarak Mandal University, Pune, IND

**Keywords:** chemotherapy-induced peripheral neuropathy (cipn), complementary therapy, neurotoxicity, quality of life (qol), yoga

## Abstract

Chemotherapy-induced peripheral neuropathy (CIPN) is a frequent complication of cancer treatment that causes sensory symptoms and emotional distress that impair the quality of life (QOL). Current treatments often fall short, necessitating innovative adjunct interventions. Yoga, which combines mindful movement, breathwork, and meditation, offers a holistic approach to mitigating the physical and psychosocial burdens of CIPN. Early trials suggest that yoga may reduce pain, enhance functionality, and support psychological well-being, although they are limited by small samples and inconsistent methods. Barriers to adoption include clinician skepticism, access challenges, and various research protocols. Prioritizing large-scale standardized trials, qualitative research on patient experiences, and telehealth delivery can strengthen yoga’s evidence base and accessibility. Multidisciplinary care models integrating yoga can transform CIPN management and promote patient-centered recovery in oncology.

## Editorial

Introduction

Cancer represents a significant global health challenge, with 18 million new cases and 9.6 million deaths reported in 2018, a figure that is anticipated to increase to 26 million cases by 2040 [1]. This growing burden places considerable strain on the healthcare systems worldwide. Although anticancer therapies such as chemotherapy, immunotherapy, and targeted treatments are essential, chemotherapy often results in chemotherapy-induced peripheral neuropathy (CIPN), which affects up to 70% of patients [2]. Persistent symptoms, including numbness, tingling, pain, and emotional distress, significantly diminish the quality of life (QOL) after treatment [3,4]. Conventional therapies provide limited relief, which highlights the need for innovative approaches. Yoga, an ancient practice that integrates mindful movements, breathing, and meditation, has emerged as a promising adjunctive intervention. This editorial explores the pathophysiology of CIPN, critiques standard treatments, evaluates the potential of yoga, and proposes research and clinical strategies for its integration into oncology.

CIPN: burden and impact

CIPN is a common dose-limiting side effect of chemotherapy, with incidence rates ranging from 30% to 70%, depending on agents such as platinum compounds, taxanes, and vinca alkaloids [2]. A systematic review found a prevalence of 68.1% within the first month after chemotherapy, which decreased to 30% after six months, although some patients continued to experience chronic symptoms [3]. This variability is due to differences in chemotherapy regimens and timing of assessments. Sensory loss, paresthesia, dysesthesia, and numbness can impair functionality, increase the risk of falls, and raise healthcare costs [3,4]. Patients often feel isolated because their symptoms are intangible and may be under-recognized by clinicians [4]. As cancer survivorship increases, addressing the physical and psychosocial effects of CIPN is crucial.

Pathophysiology of CIPN

CIPN results from complex mechanisms, including axonal degeneration, oxidative stress, mitochondrial toxicity, and neuroinflammation [5]. Neurotoxic drugs disrupt peripheral nerve function through DNA damage, ion channel dysregulation, and chemokine signaling, thereby perpetuating neuronal injury [3,6]. This intricate pathophysiology creates a challenging symptom profile that is resistant to conventional interventions, necessitating therapies targeting both neurological and systemic damage [5].

Current treatment landscape

Pharmacological treatments such as duloxetine and gabapentin provide modest pain relief but are limited by side effects and inconsistent efficacy [2,7]. Duloxetine offers moderate benefits, while gabapentin’s impact varies widely [7]. Non-pharmacological options, such as physical therapy and acupuncture, show promise but lack standardized evidence for widespread adoption [8]. Patients frequently express frustration with inadequate symptom management and limited clinician guidance, highlighting the need for accessible, patient-centered alternatives [4]. This therapeutic gap highlights the potential for holistic interventions like yoga to address unmet needs.

Potential role of yoga

Yoga, which encompasses physical postures, breathwork, and meditation, offers a holistic approach to mitigate CIPN’s physical and psychosocial burdens [9]. Evidence from analogous conditions, such as diabetic neuropathy, indicates that yoga can enhance circulation, promote neuroplasticity, and reduce stress mechanisms that may be applicable to CIPN [9,10]. Its affordability, accessibility, and low-risk profile render it an appealing complement to conventional therapies [10]. Preliminary studies underscored the therapeutic potential of yoga. A randomized controlled trial (RCT) conducted by Bao et al. demonstrated that an eight-week yoga program reduced pain by 1.95 points on the Numeric Rating Scale and improved functional reach, a predictor of fall risk, among 41 cancer survivors, surpassing the usual care [11]. Zhi et al. reported significant reductions in anxiety on the Hospital Anxiety and Depression Scale following a 12-week yoga program [12]. Knoerl et al. observed improvements in fatigue and depression, although pain relief was not statistically significant [13]. These findings suggest that yoga may alleviate pain, enhance functionality, and improve psychological well-being. However, small sample sizes, methodological variability (e.g., diverse control groups and inconsistent protocols), and variable adherence rates limit generalizability and necessitate cautious interpretation [11-13].

Challenges to integration

Integrating yoga into the management of CIPN presents both practical and perceptual challenges. Limited access to qualified instructors, financial constraints, and skepticism among clinicians rooted in yoga's historical classification as an alternative therapy hinders its adoption [4,14]. Patients report inadequate self-management guidance, exacerbating isolation [15,16]. Research challenges include inconsistent yoga protocols and difficulties in designing rigorous control arms [14]. Addressing these barriers requires standardized interventions, clinician education, and culturally sensitive approaches to accommodate diverse patient needs.

Future directions

Research must address the evidence gaps through rigorous, multifaceted strategies to establish yoga as a robust adjunct for CIPN. Large-scale, standardized RCTs, such as the phase III trial by Han et al. evaluating yoga’s efficacy in 268 survivors with chronic CIPN pain over eight weeks with a 24-week follow-up, are critical for enhancing the statistical power and reliability [17]. Long-term follow-up will elucidate yoga’s sustained effects on symptoms and QOL, while diverse participant cohorts spanning cancer types, stages, and demographics will ensure generalizability. 

Standardizing yoga protocols will facilitate cross-study comparison and evidence synthesis. Comparative trials pitting yoga against interventions such as duloxetine or acupuncture will delineate its relative efficacy [8]. Incorporating biomarkers could enable personalized yoga interventions tailored to individual physiological profiles [12]. Telehealth delivery offers a scalable solution to enhance accessibility, particularly for underserved populations, overcoming barriers such as geographic isolation and instructor shortages [8,17]. 

Qualitative research is vital for capturing the psychosocial impact of CIPN and patients’ lived experiences. Studies highlight the emotional burden, including feelings of being unheard due to clinicians’ limited time or awareness [15,16]. Exploring patients’ symptom interpretations, self-management strategies, and cultural contexts can inform tailored survivorship programs [16]. Multidisciplinary care models integrating oncology, neurology, and complementary therapy expertise could amplify the benefits of yoga, embedding it in holistic CIPN management [16,17]. By combining rigorous trials with patient-centered insights, research can position yoga as an empowering and scalable intervention for cancer survivors.

Conclusion

Yoga has potential as a safe and holistic adjunctive therapy for CIPN, with preliminary evidence indicating its benefits in pain management, functional improvement, and psychological well-being. Nevertheless, the limitations of small-scale studies and methodological constraints highlight the necessity for larger standardized trials and qualitative research to substantiate their efficacy and address the psychosocial needs of patients. Collaborative efforts among researchers, clinicians, and patients are crucial for overcoming integration barriers and incorporating yoga into oncology care. By advancing evidence and promoting patient-centered approaches, yoga may enhance recovery for cancer survivors facing persistent challenges of CIPN.
